# Effect of Foot Reflexology Intervention on Depression, Anxiety, and Sleep Quality in Adults: A Meta-Analysis and Metaregression of Randomized Controlled Trials

**DOI:** 10.1155/2020/2654353

**Published:** 2020-09-15

**Authors:** Wei-Li Wang, Hao-Yuan Hung, Ying-Ren Chen, Kuang-Huei Chen, Szu-Nian Yang, Chi-Ming Chu, Yuan-Yu Chan

**Affiliations:** ^1^Department of Psychiatry, Taoyuan Armed Forces General Hospital, Taoyuan, Taiwan; ^2^Department of Pharmacology, National Defense Medical Center, Taipei, Taiwan; ^3^Department of Pharmacy Practice, Tri-Service General Hospital, National Defense Medical Center, Taipei, Taiwan; ^4^Graduate Institute of Medical Sciences, National Defense Medical Center, Taipei, Taiwan; ^5^Department of Nursing, Taoyuan Armed Forces General Hospital, Taoyuan, Taiwan; ^6^Graduate Institute of Nursing, College of Nursing, Taipei Medical University, Taipei, Taiwan; ^7^Tri-Service General Hospital, Beitou Branch, National Defense Medical Center, Taipei, Taiwan; ^8^Graduate Institute of Health and Welfare Policy, School of Medicine, National Yang-Ming University, Taipei, Taiwan; ^9^Department of Epidemiology, School of Public Health, National Defense Medical Center, Taipei, Taiwan; ^10^Department of Psychology, Chung Yuan Christian University, Taoyuan, Taiwan

## Abstract

**Objectives:**

The aim of this study was to conduct a systematic review, meta-analysis, and metaregression to determine the current best available evidence of the efficacy and safety of foot reflexology for adult depression, anxiety, and sleep quality.

**Methods:**

Electronic databases (PubMed, ClinicalKey, ScienceDirect, EMBASE, PsycINFO, and the Cochrane Library) were searched till August, 10, 2020, and the validity of the eligible studies was critically appraised. Randomized controlled trials comparing foot reflexology groups with control groups for adult depression, anxiety, and sleep quality were included. Twenty-six eligible studies were included to assess the effect of foot reflexology intervention on the reducing symptoms of depression and anxiety and improving quality of sleep, respectively, as the primary outcome.

**Results:**

Twenty-six randomized controlled trials involving 2,366 participants met the inclusion criteria. The meta-analyses showed that foot reflexology intervention significantly improved adult depression (Hedges' *g* = −0.921; 95% CI: −1.246 to −0.595; *P* < 0.001), anxiety (Hedges' *g* = −1.237; 95% CI −1.682 to −0.791; *P* < 0.001), and sleep quality (Hedges' *g* = −1.665; 95% CI −2.361 to −0.970; *P* < 0.001). Metaregression reveals that an increase in total foot reflexology time (*P* = 0.002) and duration (*P* = 0.01) can significantly improve sleep quality.

**Conclusions:**

Foot reflexology may provide additional nonpharmacotherapy intervention for adults suffering from depression, anxiety, or sleep disturbance. However, high quality and rigorous design RCTs in specific population, along with an increase in participants, and a long-term follow-up are recommended in the future.

## 1. Introduction

Foot reflexology is a systemic practice in which a practitioner applies some pressure to any pressure points on the feet to stimulate the body and provide health benefits to different parts of the body. Foot reflexology is commonly practiced as a complementary therapy and is one of the nonpharmacological therapies to alleviate our mental, emotional, and spiritual health, while improving the quality of our life [1].

Foot reflexology is a reflexology intervention that has been applied in different cultures around the world for thousands of years. It is defined as a type of therapy that is based on the stimulation of the nerves and circulatory system of the body in which all the reflexology points, corresponding to different parts of the human body, are considered [2]. It is still ambiguous regarding the mechanism behind the function of foot reflexology, but it certainly has been shown to have potent physiological and psychological effects, perhaps attributed to the relaxation derived from the placebo effect, the therapeutic communication techniques, and impact of touching behavior. The explication for the mechanism of action in foot reflexology is based on the theory that helps to equilibrate the energy in the whole physical structure [3, 4]. Currently, the most promising theory suggests that the benefits of foot reflexology may be caused by modulating our autonomic nervous system [5]. The effects are well known to relieve the psychological symptoms of stress by reducing anxiety and muscle tension [6], calming our mood [7], improving the quality of sleep [8], and facilitating the feeling of well-being [9]. Pharmacological treatment of prevalent symptoms such as depression, anxiety, and sleep disturbance may contribute to the high strain on the body, creating additional side effects [10]. Foot reflexology provides an advantage to certain groups and generally does not cause any damaging effects during certain medical circumstances. Every person's body circumstance is unique, so outcomes from foot reflexology intervention could differ from one person to another [11].

The previous systematic review had reported physiological and biochemical outcomes associated with foot reflexology intervention [12]. However, there are insufficient number of evidence-based studies that expound the effects of foot reflexology on improving our psychological symptoms such as depression, anxiety, and sleep disturbance. To our knowledge, this is the first systematic review and meta-analysis on the psychological effect of foot reflexology and to identify the possible related factors of foot reflexology in adult participants.

## 2. Methods

### 2.1. Reporting Standards

The present study was designed, executed, and adopted in accordance with the Preferred Reporting Items for Systematic Reviews and Meta-analyses (PRISMA) statement guidelines [13] and the suggestions by the Cochrane Collaboration [14]. The protocol for this systematic review and meta-analysis is registered with PROSPERO under registration number CRD42020162545.

### 2.2. Eligibility Criteria

#### 2.2.1. Types of Studies

Randomized controlled trials (RCTs), randomized crossover trials, and cluster randomized trials all met our inclusion criteria. The language of the studies was restricted to English.

#### 2.2.2. Types of Participants

Adults aged 18 years or older without restrictions on sociodemography, race, gender, or health status were participants. All studies that reported on depression, anxiety, or sleep quality were included. There was no restriction on the baseline for these.

#### 2.2.3. Types of Interventions

No further restrictions were made regarding the foot reflexology zone, constitution, length, frequency, or duration of intervention programs. Studies on cointerventions that included foot reflexology as a part of multimodal interventions were excluded because it would be hard to evaluate the influence of foot reflexology from additional modalities. Shame intervention, care-as-usual, nontreatment waitlists, and psychoeducation about depression, anxiety, or sleep hygiene information are considered as the nonactive control group.

#### 2.2.4. Types of Outcome Measures

Studies include, at least, one efficacy outcome index related to depression, anxiety, and sleep quality. Our primary outcome measures of this study were depression, anxiety, and sleep disturbance. Data are presented both at baseline and after intervention. We take various clinical outcomes that were informed in the selected RCTs to show improvements in the symptoms of depression, anxiety, and sleep disturbance into consideration. No restrictions were set on the scales of measurement used to evaluate these outcomes because a wide variety of measures in the outcomes were applied in the studies.

Our secondary outcome of this study was intervention safety, which assessed the number of participants with adverse events, including serious adverse events or nonserious events. Adverse events resulting in death, life-threatening situations, hospitalization, disability or permanent damage, congenital anomaly/birth defect, or the need for medical or surgical intervention to prevent the aforementioned outcomes were defined as serious [15]. All other adverse events were regarded as nonserious.

### 2.3. Search Methods

The following electronic databases were searched from their inception to August 10, 2020: PubMed, ClinicalKey, ScienceDirect, EMBASE, PsycINFO, and the Cochrane Library. The search was performed using the keywords “foot reflexology,” “depression,” “anxiety,” and “sleep quality.” The complete search through PubMed was conducted using the medical subjective headings (MeSHs) as follows: (foot reflexology [MeSH] OR foot reflexology [Title/Abstract] OR foot massage [MeSH] OR foot massage [Title/Abstract] OR reflexology [MeSH] OR reflexology [Title/Abstract]) AND (depression [MeSH] OR depression [Title/Abstract] OR depressive disorder [MeSH] OR depressive disorder [Title/Abstract] OR anxiety [MeSH] OR anxiety [Title/Abstract] OR anxiety disorder [MeSH] OR anxiety disorder [Title/Abstract] sleep quality [MeSH] OR sleep quality [Title/Abstract] OR insomnia [MeSH] OR insomnia [Title/Abstract] OR sleep disturbance [MeSH] OR sleep disturbance [Title/Abstract]). The search strategy was adapted for each database as necessary.

The references of our retrieved studies and previous systematic reviews were manually screened to ensure a comprehensive search. Additionally, Google Scholar search engine was utilized to identify extra articles that had not yet been included in the previously mentioned electronic databases.

The titles and abstracts were scanned independently by two independent reviewers. When there was disagreement on eligibility, we discussed with a third reviewer to reach a consensus.

### 2.4. Data Extraction Method

Two reviewers independently extracted general information from the aforementioned selected publications on design and study sample (e.g., article setting, first author's name/year of publication, and origin), participants (e.g., mean age, gender, clinical characteristics, comorbid conditions, and the number of participants), interventions (e.g., foot reflexology zone, components, frequency, duration, and length of foot reflexology), control interventions (e.g., shame intervention, treatment-as-usual, and waitlist), and outcomes (e.g., outcome measurement tools, measured outcomes, adherence, eventual follow-up time, and adverse events). Any disagreements between the two reviewers should refer to the third reviewer's opinion.

### 2.5. Quality and Risk-of-Bias Assessments

Two reviewers independently assessed the risk of bias in each study. There were seven domains of assessment for the risk of bias including the following: (1) random sequence generation, (2) allocation concealment, (3) blinding of participants and personnel, (4) blinding of outcome assessment, (5) incomplete outcome data, (6) selective reporting, and (7) other biases using the Cochrane Systematic Review Manual risk-of-bias assessment tool [16]. These rates were then labeled as “low risk,” “high risk,” or “unclear risk” of bias. A risk-of-bias table was completed for each included study. To improve accuracy, any disagreements would refer to a third reviewer's opinion.

### 2.6. Data Synthesis and Statistical Analysis

Meta-analysis was performed using Comprehensive Meta-Analysis Software. The random-effects model was used to calculate the pooled effect size of the included studies. Hedges' *g* was calculated to determine the effect size [17]. The effect size represents the difference between two groups in the number of standard deviations. An effect size of 0.2–0.49 was considered a small effect, 0.5–0.79 was a moderate effect, 0.8 and higher was a large effect [18]. The meta-analysis results were expressed as the pool effect, with corresponding 95% and *P* value. The heterogeneity data were evaluated using a random-effects model because it accommodated the possibility that the underlying effect differed across studies.

### 2.7. Assessment of Heterogeneity

Heterogeneity between studies was evaluated using the I^2^ statistic with a cutoff point >50%, and a *P* value ≤0.1 was regarded as a significant degree of heterogeneity. The most common *I*^2^ scale considered values lower than 25% as low heterogeneity; values between 25%–50% as mean heterogeneity; values between 50%–74% as substantial heterogeneity; and values between 75%–100% as considerable heterogeneity [14]. Random effects of univariate and multivariate meta-regressions were used to explore the source of heterogeneity if *I*^2^ > 50% and *P* value ≤0.1.

### 2.8. Moderator Analyses

We performed subgroup meta-analysis and metaregression analysis to examine possible sources of heterogeneity and survey the possible confounding effects of clinical variables. The subgroup analysis produced prespecified covariates, including outcome measurement instruments, study quality, and participant details. Additionally, continuous covariates were obtained from the metaregression analysis to investigate whether relationships were linear and consistent with the results of the categorical analysis. A metaregression model was performed to test between-subgroup interaction, and a *P* value <0.05 indicated a significant difference.

### 2.9. Risk of Publication Bias

Publication bias was explored if there were up to ten eligible trials included in the meta-analysis. Funnel plots generated using Comprehensive Meta-Analysis Software were estimated from individual studies against each study's standard error. The presence of asymmetry with a visual inspection in funnel plots was considered potentially indicative of publication bias [19]. Potential publication bias was tested using the calculation of Egger's regression method, with *P* values <0.05 suggesting the presence of bias.

## 3. Results

### 3.1. Research Material

**Figure 1:**
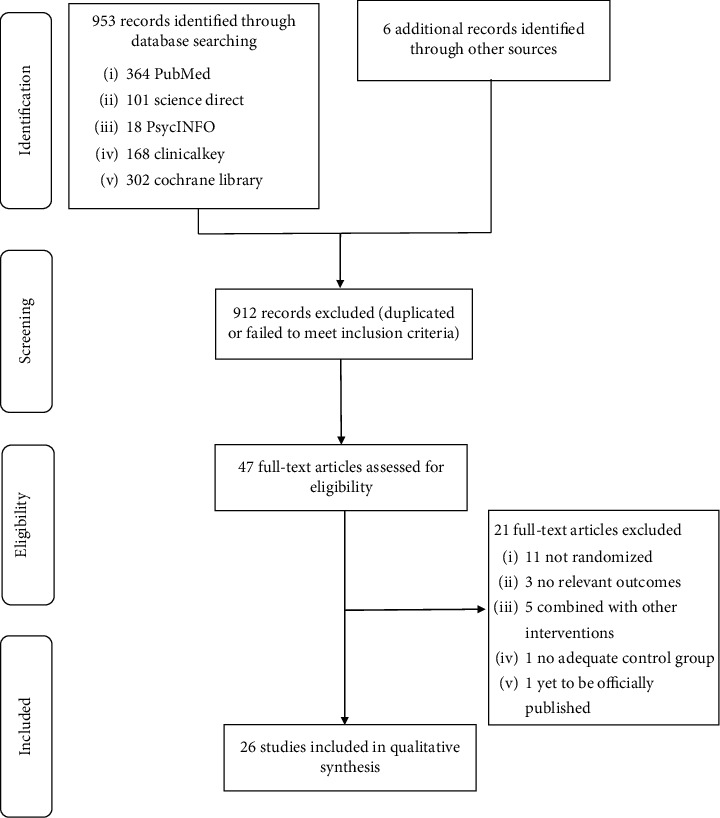
Flowchart of the results of the literature search.

The search strategy identified 959 research articles through electronic databases. A total of 912 records were excluded after removal of duplicates and screening of abstracts and titles. Then, all full-text articles were evaluated for eligibility, and 21 records were excluded for reasons such as they were not randomized [20–30], did not include relevant outcomes [31–33], included foot reflexology as a part of a multimodal intervention [34–38], lacked adequate control group [39], has yet to be officially published [40]. Finally, 26 remaining articles with 2,366 participants were investigated by qualitative analyses. All articles were published in English. The flowchart of the study selection process is presented in Figure 1.

### 3.2. Characteristics of Eligible Studies

The characteristics of the 26 eligible studies are presented in Tables 1 and 2. All study assessed outcomes are listed directly at the end of foot reflexology intervention. Among the 26 RCTs selected for our study efficacy, the psychological symptoms of depression, anxiety, and sleep quality, respectively, were assessed as the primary outcome. Our studies were conducted in Iran, Turkey, Taiwan, South Korea, Japan, and Israel. All of the included studies were published between 2011 and 2020. The sample sizes ranged from 50 to 189 individuals (total 2,366 participants). The average age ranged from 27 to 72 years, and all the participants were adults (age > 18 years). In each study, foot reflexology intervention for one session lasted between 10–60 min (total treatment sessions ranged from 1 to 18 in each study). Also, the total treatment periods ranged from 1 to 8 weeks. Adherence to the foot reflexology was reported in all studies as the percentage in foot reflexology session dropouts. Adherence was >90% in all studies. Evidence of safety issue evaluation was limited because only a few studies report safety-related adverse effects as the secondary outcome. Most of the included studies failed to report on this aspect making research difficult.

### 3.3. Risk of Bias

#### 3.3.1. Quality of Methods

Risk-of-bias assessment is shown in Table 3. Twenty-six studies were assessed as high or unclear risk of bias in at least one of the domains. All studies reviewed stated they were randomized, whether or not this is true remains uncertain as eight studies did not show their content and method of random sequencing [7, 41–47]. A small proportion of studies were low risk due to the state of detailed randomization and allocation methods [20, 48–52]. Most studies yielded no data material on bias concealment. One study had insufficient data on attrition rates [44]. We found no included studies with potential bias in the domain of selective reporting. Other potential sources of bias were high in 9 RCTs due to poor compliance, incomplete outcome data, small sample size, or obvious baseline differences [41–46, 48, 52, 53].

#### 3.3.2. Publication Bias

**Figure 2:**
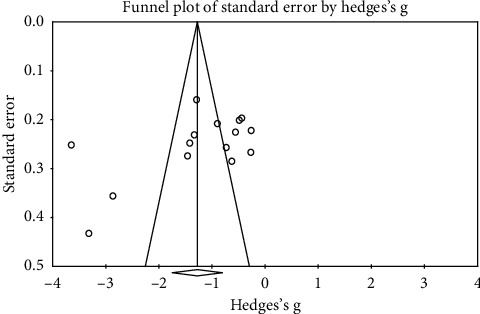
Visual inspection of the funnel plot for effect for improving anxiety symptom.

The funnel plots on the efficacy of foot reflexology for psychological symptoms for depression, anxiety, and sleep disturbance were executed including 4 RCTs, 16 RCTs, 10 RCTs, respectively. The visual inspection of the funnel plots indicated some risk of publication bias for the effects of foot reflexology only in the domain of anxiety symptoms (shown in Figure 2). Those plots examined were shown to be asymmetrical, suggesting the possible risk of publication bias. Moreover, results of Egger's regression test indicated no significant publication bias (Egger test intercept = −7.32; *P* = 0.11). Therefore, the overall population effect size was likely to be relatively robust.

### 3.4. Efficacy Analysis (Results from Each Meta-Analysis) Primary Outcomes

The sizes of effect for selected studies were prominent in depression, anxiety, and sleep disturbance. The data revealed in Table 4 that foot reflexology intervention resulted in significant improvement in adults with depression, anxiety, and sleep problems.

#### 3.4.1. Depression

**Figure 3:**
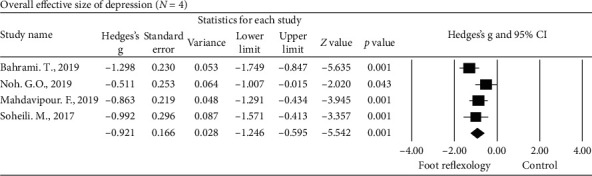
Overall effect size of the improvement of depression in adults following foot reflexology intervention (*n* = 4 studies).

Four studies [7, 50, 54, 55] investigated depression as the primary outcome following foot reflexology intervention by using different depression outcome measurement tools. These tools included the Beck Depression Inventory Scale; hospital anxiety and depression scale; and depression, anxiety, and stress scale-21 and were used in our meta-analysis. Hedges' *g* for the overall effect size was −0.921, and the 95% CI was −1.246 to −0.595 (Figure 3). The sample collection sizes of effect for sample collection all came out negative, with Hedges' *g* ranging from −0.511 to −1.298. Reviewing the results, it is clear that there was significant reduction in depression following foot reflexology intervention, with a large effect size. There was mean heterogeneity among the studies of depression (*Q* = 5.42, *P* = 0.143, *I*^2^ = 44.74).

#### 3.4.2. Anxiety

**Figure 4:**
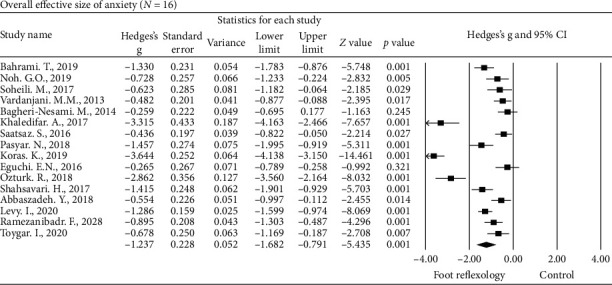
Overall effect size of the improvement of anxiety in adults following foot reflexology intervention (*n* = 16 studies).

The sixteen studies [45–48, 50, 52–62] examined anxiety as the primary outcome following foot reflexology intervention by using different anxiety outcome measurement tools such as the hospital anxiety and depression scale; depression, anxiety, and stress scale-21; State-Trait Anxiety Inventory; and Visual Analogue Scale of Anxiety and were included in our meta-analysis. Hedges' *g* for the overall effect size was −1.237, and the 95% CI was −1.682 to −0.791 (Figure 4). The sample collection effect sizes all came out negative, with Hedges' *g* ranging from −0.259 to −3.644. These results suggested that the overall reduction in anxiety following foot reflexology intervention was significant, with a large effect size. Heterogeneity among the studies of anxiety was considerably large (*Q* = 217.41, *P* < 0.001, *I*^2^ = 93.10).

#### 3.4.3. Sleep Quality

**Figure 5:**
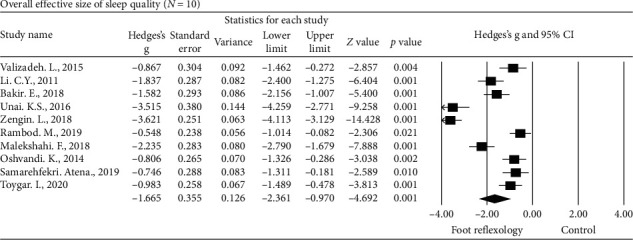
Overall effect size of the improvement of sleep quality in adults following foot reflexology intervention (*n* = 10 studies).

Hedges' *g* of the ten studies [8, 41–44, 49, 51, 62–64] examined sleep quality following foot reflexology intervention by using different outcome measurement tools such as the Pittsburgh Sleep Quailty Index; the Verran and Snyder-Halpern Sleep Scale; St. Mary's Hospital Sleep Questionnaire; and Richard–Campbell Sleep Questionnaire which were included in our meta-analysis. Hedges' *g* for the overall effect size was −1.665, and the 95% CI was −2.361 to −0.970 (Figure 5). The effect sizes for sample collection were unsurprisingly all negative, with Hedges' *g* ranging from −0.548 to −3.621. The meta-analysis revealed that the overall improvement in sleep quality following foot reflexology intervention was significant, with a large effect size. Considerable heterogeneity was observed among the studies in sleep quality where the outcomes measured (*Q* = 144.87, *P* < 0.001, *I*^2^ = 93.78).

Substantial heterogeneity was found in the anxiety and sleep quality studies. Therefore, subgroup analyses along with moderator and metaregression analyses were conducted to further explore the determinations of the heterogeneity.

### 3.5. Secondary Outcomes (Safety)

No adverse events were reported in the few RCTs on foot reflexology intervention for depression, anxiety, and sleep quality. Most of the included studies failed to report this aspect. Dropouts were not treated as adverse events not only because they were not explicitly explaining their personal reasons for dropout in the original study but also because our research material lacked subject commentary.

### 3.6. Subgroup Analyses and Metaregression Analyses of Anxiety and Sleep Quality

Subgroup analyses and metaregression analyses to investigate any possible confounding clinical variables within the studies are presented in Table 5.

#### 3.6.1. Anxiety

Four RCTs revealed evidence for the effects of foot reflexology when compared with the control group in reducing the anxiety level before adult undergoing coronary angiography (Hedges' *g* = −1.426, 95% CI was −2.278 to −0.575, *P* < 0.001). Two RCTs revealed evidence for the effects of foot reflexology compared with the control group in reducing the anxiety level for delivering women (Hedges' *g* = −0.869, 95% CI was −1.702 to −0.869, *P* = 0.041). Significant subgroup differences were identified for the outcome measures (STAI vs. Others; Hedges' *g* = −1.534 vs. −0.894, *P* < 0.001). Our subgroup analysis performed one session of foot reflexology intervention, before or after interventional surgery, which would be more effective than numerous sessions of foot reflexology intervention, as according to other interventional surgeries or procedure studies (one session vs. numerous sessions; Hedges' *g* = −1.553 vs. −0.849, *P* < 0.001). Other subgroup analysis indicated cardiovascular surgery or an interventional procedure was less effective than other surgery or interventional procedures (cardiovascular vs. other surgery; Hedges' *g* = −1.060 vs. −2.340, *P* < 0.001), which significantly reduced the anxiety level of psychological symptoms. The selection bias including random sequence generation and allocation concealment of study also showed significant differences in interactions between subgroups (*P* < 0.05).

In the exploratory metaregression analysis of anxiety, no significant relationship was observed between the effect size for mean age (*P* = 0.852) and total length of intervention in one time period (*P* = 0.903).

#### 3.6.2. Sleep Quality

Subgroup analysis was performed using the parameters study group and participants type. However, results of the subgroup analysis indicated that heterogeneity may have resulted from the abovementioned factors. While performing the metaregression, the mean age of participants, duration of intervention sessions, and total foot reflexology intervention time were required as possible moderating variables. The selection bias including random sequence generation and allocation concealment of study also showed significant differences in interactions between subgroups (*P* < 0.05).

Regression analyses revealed a positive correlation with the total length of foot reflexology intervention time (*P* = 0.002) and duration of intervention sessions (*P* = 0.01), indicating that the more the total length of foot reflexology intervention time and duration of intervention sessions, the more likely it is to have significant results. However, the mean age of participants did not report any significant impact (*P* = 0.897).

## 4. Discussion

### 4.1. Summary of Evidence

We analyzed the impact on foot reflexology on depression, anxiety, and sleep quality. Meta-analysis for improvement of psychological symptoms indicated that the foot reflexology could effectively relieve depression, anxiety, and sleep quality. However, effect sizes of various studies were heterogeneous. In addition, not only did we focus on the possible moderating clinical factors but also investigated the possible confounding effect by the different measurement tools.

Overall, the application of foot reflexology was not associated with degradation of psychological symptoms or a rapid increase in adverse effects. Only a few studies explicitly assessed safety-related, nonserious adverse events. Foot reflexology is most likely a comparatively safe practice for this population. However, future RCTs should take more measures to establish even more accurate reporting of adverse events and personal reasoning for dropouts from participants.

### 4.2. Comparison with Prior Reviews

No systematic review explicitly focusing on foot reflexology for improving psychological symptoms including depression, anxiety, and sleep quality was accessible. Ours is the first systematic review and meta-analysis with 26 RCTs to focus on the effects of foot reflexology on depression, anxiety, and sleep quality. We identified there is no direct correlation or evidence on previous meta-analysis reports on self-administered foot reflexology with subjective and objective outcomes for healthy persons [65], benefits of foot reflexology for insomnia [66], or effects of foot reflexology on fatigue, sleep, and pain [67]. The studies of these analyses are nonrandomized trials before-and-after studies, and the sample size of these studies was too small. Future research should ensure detailed and precise methodology and adequate sample size to better evaluate the impact of foot reflexology intervention. Results of previous reviews published on 2019 reveal effectiveness of reflexology intervention on premenstrual syndrome [68] and anxiety of patients undergoing cardiovascular interventional procedures [69]. These two recent reviews illustrated that all reflexology intervention practices including hand reflexology and foot reflexology benefited participants in specific groups. Our meta-analysis with 26 RCTs emphasized foot reflexology intervention on depression, anxiety, and sleep quality and conducted further exploration on the determinants of the heterogeneity with subgroup analysis for both categorical and continuous moderators to find significant factors for perceived heterogeneity.

### 4.3. External and Internal Validity

Major threats to external validity included specific variables of sampled participants and multiple foot reflexology intervention types. The majority of RCTs included participants from Asia. The lack of studies from America, Europe, and Africa was apparent. It might not be applicable to other areas. Heterogeneity is high due to wide variability in participant groups, foot reflexology technique, selection of reflexology zones, foot reflexology intervention duration, and frequency.

Internal validity is limited due to the methodological quality of the included studies. All of the included studies used self-reported questionnaires for depression, anxiety, and sleep quality; thus, recall bias could not be excluded. It remains to be determined whether differences in these parameters could affect results. All of our studies asserted that they had applied randomization methods; however, not all of the studies elaborate on the design protocol and methods of randomization, and some of the included studies seem to not have been truly randomized. It also proves difficult to properly blinding. Only one of the reviewed RCTs successfully implemented blinding in the participants [62]. Erroneous random sequence generation and allocation concealment have been empirically revealed to be a significant source of bias in RCTs [70]. Our included studies only had a low risk or an unclear risk of selection bias with no high risk selection bias. All the effects were robust against potential risk of selection bias, and the internal validity of the review, while limited, is still acceptable.

### 4.4. Strengths and Weaknesses

This is the first and latest comprehensive systematic review and meta-analysis available on foot reflexology for depression, anxiety, and sleep quality with a large number of randomized controlled trials. None of studies provided any adverse effects of foot reflexology, thereby indicating the importance of using foot reflexology as an effective and less complicated intervention practice. There were seven primary limitations of this review [71]. First, despite great efforts to locate all relevant RCTs of foot reflexology intervention for psychological symptoms, there may be a degree of uncertainty due to a limitation in interlanguage communication, limited resources, and bias in publication. Due to language constraints, we did not include Arab States, Japanese, and Korean database. Second, only one of the studies provided the methods of blinding. Participant blinding is sometimes impossible to fully control; for example, trials in sport, surgical intervention, nonpharmacological therapy, were all not valued as appropriate, lacking pragmatic and systemic aim. Previous studies provided empirical evidence of pronounced bias due to deficiency in patient bias control in related randomized clinical trials with patient-reported outcomes [72]. Third, the critical flaw of this study was the relative lack of high-quality RCTs. The small number of participating studies meant that the statistical power to detect differences was suboptimal. Future large-scale trials may be recommended to demonstrate this effect. Fourth, masseuses often chat with their clients which has a psychological effect which may influence this research. Social interaction has been known to reduce stress and anxiety. If some practitioners speak to their clients while others do not, this would impact results greatly. Control over social interaction is needed for further research. The fifth limitation is the severity of the complaints concerning psychological symptoms and health status of the participants. This was not considered appropriate and was not individually listed in each study. Differences in self-reported questionnaires were found between intervention and control groups in some studies. This may have led to heterogeneity. The sixth limitation was that the intensity (size of strength), frequency (sessions of per week), and duration (time of each session) of foot reflexology interventions were all heterogeneous. Most of the studies were short-term applications without long-term follow-up effects. Lastly, a lack of priority in safety evaluation may have caused each study to produce minimal occurrences in serious adverse events or nonserious events. It only can be assumed that foot reflexology intervention is a low-risk treatment option.

### 4.5. Implications for Further Research

If possible, we should expand research parameters to include western countries such as Canada or the United States. Different countries may include foot reflexology under their national health insurance or private healthcare plans. If a client has free access to this treatment, they naturally are more inclined to continue (adherence rate would increase). However, if foot reflexology is not covered under a client's healthcare provided, they would be less likely to continue (adherence rate would decrease). This systematic review and meta-analysis were limited by the low methodological quality of included studies. Further RCTs should enforce thorough methodology and reports, which would mean appropriate sample size, adequate randomization, allocation concealment, intention-to-treat analysis, and bias control of the least one outcome assessors [73]. In order to achieve successful bias control of participants and minimize any physiological effects, a physical force less than the minimal force is required in foot reflexology at nonreflexology areas and may be regarded as a sham control. According to the funnel plots, there could be a publication bias in which authors lose confidence in their published trials if their results produced negative conclusions. The quality of the results of meta-analysis was determined by the quality of the RCT and by sufficient clinical evidence. Thus, if we want to draw a reasonable conclusion for a meta-analysis, we need larger sample sizes and more rigorously randomized controlled trials. Researchers for study interventions may need to apply a standard protocol to specific demographic group. Objective psychological symptoms measuring tools, such as actigraphy or heart rate variability analysis, should be incorporated to more accurately evaluate the effect of foot reflexology. There is a lack of evidence in follow-up effects of foot reflexology in psychological symptoms. So, long-term follow-ups should be necessary in future RCTs. Ample reporting of safety issues with foot reflexology intervention should be utilized in future randomized controlled trials. Limited evidence impaired our research because no studies reported safety-related adverse effects. Most of the included studies failed to report on this aspect.

## 5. Conclusions

Results of this systematic review and meta-analysis demonstrated that foot reflexology intervention has benefits compared to nonactive control practices in terms of ameliorate the burden of depression, anxiety, and sleep disturbance. Furthermore, metaregression reveals that an increase in total foot reflexology time would decrease anxiety and improve sleep quality. Despite certain flaws in methodology in our included studies, foot reflexology may be recommended as a complementary intervention to improve our depression, anxiety, and sleep quality. However, advanced strength of evidence with future understanding of the mechanisms of foot reflexology and long-term follow-up should be a priority for future preparation and implementation for sensitive groups, such as delivering women or cancer patients, who may be unable to use other means of care and benefit from such care.

## Figures and Tables

**Table 1:** Characteristics of included studies.

Authors, year, country	Main characteristics of studied population	Sample characteristics (sample size, mean age)	Sex difference	Intervention group. vs. comparison group	Outcome measurement tools	Outcomes
Valizadeh et al., 2015, Iran	Participants between the age of 60–75 y/o independently performing daily activities and having mental health based on health records available in the health center	69, G1 = 23, G2 = 23, G3 = 23 mean age: G1 = 66.82 y/o (SD = 4.80), G2 = 67.69 y/o (SD = 4.28), G3 = 66.82 y/o (SD = 3.84)	Male: 69 Female: 0	G1 = foot reflexology G2 = footbath G3 = control group	Pittsburgh Sleep Quality Index (PSQI)	The total score of PSQI improved: no statistically significant finding G1 vs. G3 (*P* < 0.05) G1 = 6.08 (5.27), G1^*∗*^ = 3.91 (4.04) G3 = 4.69 (0.51), G3^*∗*^ = 5.69 (3.08)

Lee et al., 2011, Taiwan	Postpartum women have given birth vaginally without postpartum complications and concurrent medical conditions with poor sleep condition (PSQI ≥ 5)	68, G1 = 34, G2 = 34 mean age: G1 = 32.0 y/o (SD = 2.8), G2 = 31.2 y/o (SD = 2.8) (3 drop out)	Male: 0 Female: 68	G1 = foot reflexology G2 = control group	Pittsburgh Sleep Quality Index (PSQI)	The total score of PSQI improved: G1 vs. G2 (*P* < 0.001) G1 = 9.94 (2.61), G1^*∗*^ = 3.97 (1.26) G2 = 9.45 (2.59), G2^*∗*^ = 6.24 (1.68)

Bakir et al., 2018, Turkey	Voluntary participants aged ≥18 y/o diagnosed with rheumatoid arthritis, at least, 1 year with VAS-Pain (visual analogue scale for pain) of 4 or greater	60, G1 = 30, G2 = 30 mean age: G1 = 50.83 y/o (SD = 12.0), G2 = 49.50 y/o (SD = 16.4) (5 drop out)	Male: 14 Female: 46	G1 = foot reflexology G2 = control group	Pittsburgh Sleep Quality Index (PSQI)	The total score of PSQI improved: G1 vs. G2 (*P* = 0.001) G1 = 16.20 (3.70), G1^*∗*^ = 13.16 (3.57) G2 = 16.75 (3.64), G2^*∗*^ = 19.03 (3.05)

Unal et al., 2016, Turkey	Patients between the age of 18–60 y/o who received hemodialysis therapy twice a week without any communication problems	105, G1 = 35, G2 = 35, G3 = 35 mean age: G1 = 51.74 y/o (SD = 12.2), G2 = 53.89 y/o (SD = 13.1), G3 = 54.33 y/o (SD = 12.9)	Male: 55 Female: 50	G1 = foot reflexology G2 = back massage G3 = control group	Pittsburgh Sleep Quality Index (PSQI)	The total score of PSQI improved: G1 vs. G3 (*P* < 0.05) G1 = 11.09 (3.18), G1^*∗*^ = 5.54 (2.15) G3 = 9.20 (2.42), G3^*∗*^ = 11.88 (2.47)

Zengin et al., 2018, Turkey	Participants with cancer have received at least their first session of chemotherapy and have no diagnosis of sleep disorder	167, G1 = 84, G2 = 83 mean age: G1 = not mentioned G2 = not mentioned (9 drop out)	Male: 78 Female: 89	G1 = foot reflexology G2 = control group	Pittsburgh Sleep Quality Index (PSQI)	The total score of PSQI improved: G1 vs. G2 (*P* < 0.001) G1 = 12 (2.7), G1^*∗*^ = 5.5 (2.1) G2 = 11.3 (1.9), G2^*∗*^ = 13 (2.4)

Rambod et al., 2019, Iran	Patients with lymphoma aged ≥18 y/o, being able to speak Persian and being willing to participate in the study	72, G1 = 36, G2 = 36 mean age: G1 = 41.47 y/o (SD = 13.70), G2 = 46.90 y/o (SD = 15.40)	Male: 52 female: 20	G1 = foot reflexology G2 = control group	Pittsburgh Sleep Quality Index (PSQI)	The total score of PSQI improved: G1 vs. G2 (*P* < 0.05) G1 = 10.11 (3.26), G1^*∗*^ = 8.41 (2.98) G2 = 11.80 (3.83), G2^*∗*^ = 11.83 (3.26)

Malekshahi et.al., 2018, Iran	Patients between the age of 18–65 y/o who have sleeping problems on the basis of the Pittsburgh questionnaire, undergoing hemodialysis in the evening and night shifts	80, G1 = 40, G2 = 40 mean age: G1 = not mentioned G2 = not mentioned	Male: 53 female: 27	G1 = foot reflexology G2 = control group	Pittsburgh Sleep Quality Index (PSQI)	The total score of PSQI improved: G1 vs. G2 (*P* < 0.05) G1 = 11.79 (3.13), G1^*∗*^ = 6.32 (1.93) G2 = 10.94 (4.10), G2^*∗*^ = 12.47 (3.94)

Oshvandi et al., 2014, Iran	Patients between the age of 30–80 y/o who have ischemic heart disease hospitalized in the critical care unit	60, G1 = 30, G2 = 30 mean age: G1 = 64.17 y/o (SD = 12.04), G2 = 50.50 y/o (SD = 11.40)	Male: 34 Female: 26	G1 = foot massage G2 = control group	St. Mary's Hospital Sleep Questionnaire (SMHSQ)	The total score of SMHSQ improved: G1 vs. G2 (*P* < 0.05) G1 = 19.67 (6.25), G1^*∗*^ = 15.33 (4.87) G2 = 18.93 (5.87), G2^*∗*^ = 18.90 (5.66)

Samarehfekri et al., 2020, Iran	Patients undergoing kidney transplantation surgeries suffer from postoperative pain, fatigue, and sleep disorders	50, G1 = 25, G2 = 25 mean age: G1 = 38.12 y/o (SD = 12.87), G2 = 38.56 y/o (SD = 12) (3 drop out)	Male: 34 Female: 16	G1 = foot massage G2 = control group	The Verran and Snyder-Halpern Sleep Scale	The total score of the Verran and Synder-Halpern Sleep Scale improved: G1 vs. G2 (*P* < 0.05) G1 = 41.98 (SD = 13.92), G1^*∗*^ = 60.60 (SD = 10.75) G2 = 42.15 (SD = 11.78), G2^*∗*^ = 52.23 (SD = 11.76)

Toygar et al., 2020, Turkey	Aged 18 years and above, who are the primary informal caregivers of cancer patients (without any professional help)	66, G1 = 33, G2 = 33 mean age: G1 = 41.52 y/o (SD = 13.88), G2 = 39.02 y/o (SD = 12.80)	Male: 10 Female: 56	G1 = foot reflexology G2 = control group (shame intervention)	Richard–Campbell Sleep Questionnaire (RCSQ) state-trait anxiety inventory (STAI)	The total score of RCSQ improved: G1 vs. G2 (*P* < 0.05) G1 = 430.3 (SD = 43.46), G1^*∗*^ = 441.8 (SD = 35.51) G2 = 441.2 (SD = 35.18), G2^*∗*^ = 409.5 (SD = 50.08) the total score of STAI improved: G1 vs. G2 (*P* < 0.05) G1 = 46.67 (SD = 7.21), G1^*∗*^ = 38.91 (SD = 5.63) G2 = 47.94 (SD = 10.62), G2^*∗*^ = 46.30 (SD = 11.29)
Bahrami et al., 2019, Iran	A female patient aged ≥60 y/o diagnosed with acute coronary syndrome consisting of angina pectoris and myocardia infraction, no anxiolytics and sedative medications in the last four hours before the intervention	90, G1 = 45, G2 = 45 mean age: G1 = 72.86 y/o (SD = 7.98), G2 = 72.62 y/o (SD = 7.93)	Male: 0 Female: 90	G1 = foot reflexology G2 = control group	Hospital depression scale (HADS-D) hospital anxiety scale (HADS-A)	The total score of HADS-D improved: G1 vs. G2 (*P* < 0.05) G1 = 13.66 (SD = 4.64), G1^*∗*^ = 8.42 (SD = 3.62) G2 = 11.74 (SD = 4.29), G2^*∗*^ = 11.11 (SD = 3.42) the total score of HADS-A improved: G1 vs. G2 (*P* < 0.05) G1 = 13.77 (SD = 4.39), G1^*∗*^ = 8.53 (SD = 3.71) G2 = 11.66 (SD = 4.24), G2^*∗*^ = 11.06 (SD = 3.19)

Noh et al., 2019, South Korea	Gynaecologic cancer patients receiving chemotherapy and hospitalized in the gynaecological ward, who received short-term chemotherapy (at least 2 weeks chemotherapy)	63, G1 = 32, G2 = 31 mean age: G1 = 56.34 y/o (SD = 9.04), G2 = 55.36 y/o (SD = 9.96) (1 drop out)	Male: 0 Female: 63	G1 = self-foot reflexology G2 = control group	Hospital depression scale (HADS-D); hospital anxiety scale (HADS-A)	The total score of HADS-D improved: G1 vs. G2 (*P* < 0.01) G1 = 9.31 (SD = 4.47), G1^*∗*^ = 8.03 (SD = 4.28) G2 = 8.58 (SD = 4.36), G2^*∗*^ = 9.48 (SD = 4.14) the total score of HADS-A improved: G1 vs. G2 (*P* < 0.01) G1 = 7.25 (SD = 4.05), G1^*∗*^ = 5.69 (SD = 3.46) G2 = 6.48 (SD = 3.06), G2^*∗*^ = 7.39 (SD = 3.23)

Mahdavipour et al., 2019, Iran	Women during their menopausal period, aged 40–60 y/o, diagnosis of depression by a psychiatrist based on DSM-IV, and the total depression score >14 based on the Beck Depression Inventory	90, G1 = 45, G2 = 45 mean age: G1 = 54.18 y/o (SD = 3.90), G2 = 52.23 y/o (SD = 11.6) (10 drop out)	Male: 0 Female: 90	G1 = foot reflexology G2 = control group	Beck Depression Inventory-second edition (BDI-II)	The total score of BDI-II improved: G1 vs. G2 (*P* < 0.001) G1 = 26.97 (SD = 4.47), G1^*∗*^ = 22.55 (SD = 5.18) G2 = 26.15 (SD = 5.01), G2^*∗*^ = 26.22 (SD = 5.14)

Soheili et al., 2017, Iran	Female patients aged 18–75 y/o, with a definite diagnosis of multiple sclerosis by a medicine specialist	75, G1 = 25, G2 = 25, G3 = 25 mean age: G1 = 34.4 y/o (SD = 6.6), G2 = 33.9 y/o (SD = 5.6) G3 = 34.0 y/o (SD = 7.7)	Male: 0 Female: 75	G1 = foot reflexology G2 = relaxation G3 = control group	Depression, anxiety and stress scale‐21 (DASS‐21)	The total score of DASS-21 depression improved: G1 vs. G3 (*P* = 0.03) G1 = 20.72 (SD = 7.56), G1^*∗*^ = 13.20 (SD = 6.16) G3 = 19.52 (SD = 6.06), G3^*∗*^ = 18.64 (SD = 6.99) the total score of DASS-21 anxiety improved: G1 vs. G3 (*P* = 0.03) G1 = 16.72 (SD = 6.66), G1^*∗*^ = 10.40 (SD = 7.37) G3 = 16.80 (SD = 6.90), G3^*∗*^ = 14.88 (SD = 6.50)

Vardanjani et al., 2013, Iran	The patients were candidates for their first elective coronary angiography without the symptoms of myocardial infarction	100, G1 = 50, G2 = 50 mean age: G1 = 52.6 y/o (SD = 7.8), G2 = 54.8 y/o (SD = 5.6)	Male: 100 female: 0	G1 = foot reflexology G2 = control group	State-Trait Anxiety Inventory (STAI)	The total score of STAI improved: G1 vs. G2 (*P* = 0.0001) G1 = 53.24 (SD = 4.29), G1^*∗*^ = 45.24 (SD = 3.32) G2 = 49.62 (SD = 5.31), G2^*∗*^ = 43.70 (SD = 5.06)

Bagheri-nesami et al., 2014, Iran	Voluntary participants participate in the study for first nonemergency cardiac surgery by using a heart-lung machine	80, G1 = 40, G2 = 40 mean age: G1 = 58.75 y/o (SD = 8.69), G2 = 58.90 y/o (SD = 9.58)	Male: 40 female: 40	G1 = foot reflexology G2 = control group	Visual Analogue Scale of Anxiety (VAS-A)	The total score of VAS-A improved: G1 vs. G2 (*P* < 0.05) G1 = 1.93 (SD = 2.81), G1^*∗*^ = 1.45 (SD = 2.90) G2 = 1.78 (SD = 2.11), G2^*∗*^ = 2.00 (SD = 2.44)

Khaledifar et al., 2017, Iran	Participants aged ≥18 y/o, candidate for coronary angiography in hospital, absence of acute psychological disorders, or use of antistress drugs within recent 48 hours	75, G1 = 25, G2 = 25, G3 = 25 mean age: G1 = 67.2 y/o (SD = 11.8), G2 = 67.0 y/o (SD = 11.1) G3 = 64.7 y/o (SD = 12.1)	Male: 38 female: 37	G1 = foot reflexology G2 = massage therapy G3 = control group	State-Trait Anxiety Inventory (STAI)	The total score of STAI improved: G1 vs. G3 (*P* < 0.05) G1 = 60.60 (SD = 7.20), G1^*∗*^ = 34.70 (SD = 4.70) G3 = 47.80 (SD = 9.60), G3^*∗*^ = 46.50 (SD = 9.20)

Saatsaz et al., 2016, Iran	Female, aged 20–35 y/o, being primiparous, giving birth to a living and healthy child, being conscious, and having junior high school or higher degree of education to comprehend the numerical pain scale	106, G1 = 52, G2 = 52, G3 = 52 mean age: G1 = 27.04 y/o (SD = 2.77), G2 = 26.73 y/o (SD = 3.81), G3 = 27.75 y/o (SD = 3.22)	Male: 0 Female: 106	G1 = foot massage G2 = foot and hand massage G3 = control group	State-Trait Anxiety Inventory (STAI)	The total score of STAI improved: G1 vs. G3 (*P* < 0.05) G1 = 31.52 (SD = 9.93), G1^*∗*^ = 28.23 (SD = 8.88) G3 = 30.17 (SD = 6.98), G3^*∗*^ = 30.38 (SD = 6.93)

Pasyar et al., 2018, Iran	Patients who had undergone tibial shaft fracture surgery; aged ≥18 y/o; an open reduction and internal fixation surgery for a tibial fracture, hospital admission for at least 1 day after surgery	66, G1 = 33, G2 = 33 G1 = not mentioned G2 = not mentioned	Male: 53 female: 13	G1 = foot reflexology G2 = control group	State-Trait Anxiety Inventory (STAI)	The total score of STAI improved: G1 vs. G2 (*P* < 0.05) G1 = 54.72 (SD = 7.36), G1^*∗*^ = 42.84 (SD = 6.50) G2 = 57.48 (SD = 9.14), G2^*∗*^ = 58.36 (SD = 10.37)

Koras et al., 2019, Turkey	Patients age ≥18 y/o who underwent laparoscopic cholecystectomy without any complication with pain severity greater than 4 on VAS (visual analogue scale) after surgery	167, G1 = 85, G2 = 82 mean age: G1 = not mentioned G2 = not mentioned	Male: 50 female: 117	G1 = foot massage G2 = control group	State-Trait Anxiety Inventory (STAI)	The total score of STAI improved: G1 vs. G2 (*P* < 0.05) G1 = 49.74 (SD = 13.54), G1^*∗*^ = 28.67 (SD = 9.12) G2 = 43.67 (SD = 8.11), G2^*∗*^ = 51.84 (SD = 6.61)

Eguchi et al., 2016, Japan	Men and women aged 20 to 70 who lived in or near Matsuyama, Ehime Prefecture, Japan	55, G1 = 27, G2 = 28 mean age: G1 = 49.0 y/o (SD = 13.6), G2 = 48.8 y/o (SD = 11.4)	Male: 5 female: 50	G1 = foot reflexology G2 = control group	State-Trait Anxiety Inventory (STAI)	The total score of STAI improved: G1 vs. G2 (*P* < 0.05) G1 = 41.1 (SD = 11.2), G1^*∗*^ = 38.0 (SD = 9.4) G2 = 40.6 (SD = 10.0), G2^*∗*^ = 40.0 (SD = 9.2)

Ozturk et al., 2018, Turkey	Voluntary participants who have undergone abdominal hysterectomy operation and reported postoperation pain of 3 or above according to visual analog scale	63, G1 = 32, G2 = 31 mean age: 47.23 y/o (SD = 4.71)	Male: 0 female: 63	G1 = foot reflexology G2 = control group	State-Trait Anxiety Inventory (STAI)	The total score of STAI improved: G1 vs. G2 (*P* < 0.05) G1 = 58.87 (SD = 4.81), G1^*∗*^ = 45.75 (SD = 4.25) G2 = 57.32 (SD = 4.81), G2^*∗*^ = 55.96 (SD = 3.85)

Ramezanibadr et al. 2018 Iran	Male candidates for undergoing coronary angiography, aged 40–80 y/o, had neither health problems nor arterial line in the feet, received no anxiolytic agent during the past 48 hours before the intervention	150, G1 = 50, G2 = 50, G3 = 50 mean age: 66.5 y/o (SD = 4.6)	Male: 150 Female: 0	G1 = foot reflexology G2 = placebo group G3 = control group	State-Trait Anxiety Inventory (STAI)	The total score of STAI improved: G1 vs. G3 (*P* < 0.05) G1 = 61.68 (SD = —), G1^*∗*^ = 45.58 (SD = —) G2 = 60.52 (SD = —), G2^*∗*^ = 59.14 (SD = —)

Shahsavari et al., 2017, Iran	Patients between the age of 18–60 y/o, no lesion or disorder on the feet and other conditions affecting the feet, no previous history of bronchoscopy, or participation in similar studies	80, G1 = 40, G2 = 40 mean age: G1 = 45.55 y/o (SD = 1.78), G2 = 48.23 y/o (SD = 1.72)	Male: 41 female: 39	G1 = foot reflexology G2 = control group	Visual Analogue Scale of Anxiety (VAS-A)	The total score of VAS-A improved: G1 vs. G2 (*P* < 0.05) G1 = 4.35 (SD = 2.08), G1^*∗*^ = 2.83 (SD = 1.45) G2 = 3.78 (SD = 1.83), G2^*∗*^ = 4.88 (SD = 2.15)

Abbaszadeh et al., 2018, Iran	Participants who had been diagnosed with coronary artery disease and were candidates for nonurgent CABG (coronary artery bypass graft)	120, G1 = 40, G2 = 40, G3 = 40 mean age: G1 = 55.90 y/o (SD = 8.31), G2 = 57.32 y/o (SD = 8.62) G3 = 56.30 y/o (SD = 7.11)	Male: 120 female: 0	G1 = foot reflexology G2 = placebo group G3 = control group	Short-form of Atate-Trait Anxiety Inventory (short-form of STAI)	The total score of STAI improved: G1 vs. G3 (*P* > 0.05) G1 = 8.25 (SD = 2.71), G1^*∗*^ = 6.21 (SD = 0.82) G2 = 10.81 (SD = 2.16), G2^*∗*^ = 7.80 (SD = 2.31)

Levy et al., 2020, Israel	Women aged over 18 years, hospitalization in obstetrics ward during labor, primiparity, with moderate to severe anxiety at admission Visual Analogue Scale (VAS) ≥4	189, G1 = 99, G2 = 90 mean age: G1 = 28.6 y/o (SD = 4.4), G2 = 27.9 y/o (SD = 4.5)	Male: 0 female: 189	G1 = foot reflexology G2 = control group	Visual Analogue Scale of Anxiety (VAS-A)	The total score of VAS-A improved: G1 vs. G2 (*P* < 0.05) G1 = 7.9 (SD = 1.8), G1^*∗*^ = 5.5 (SD = 2.4) G2 = 7.9 (SD = 2.0), G2^*∗*^ = 8.6 (SD = 2.4)

BDI-II = Beck Depression Inventory-second edition; CABG = coronary artery bypass graft; DASS‐21 = depression, anxiety, and stress scale‐21; DSM-IV = Diagnostic and Statistical Manual of mental disorders, 4^th^ edition; G1 = group 1, G2 = group 2, G3 = group 3; HADS-A = hospital anxiety and depression scale-anxiety; HADS-D = hospital anxiety and depression scale-depression; PSQI = Pittsburgh Sleep Quality Index; SMHSQ = St. Mary's Hospital Sleep Questionnaire; STAI = State-Trait Anxiety Inventory; RCSQ = Richard–Campbell Sleep Questionnaire; VAS-A = visual analogue scale for anxiety.

**Table 2:** Characteristics of foot reflexology programs and outcomes assessment of studies included in meta-analysis.

Authors, year	Frequency (sessions/week)	Session length (mins/session)	Duration (weeks/study)	Number of sessions/study total length/study	Safety (adverse events)	Lasting effects and duration	Adherence rate (%)
Valizadeh et al., 2015	1	20 (total 20 min, 10 min for each foot)	6	6 (2 hours)	Not reported	Not reported	23/23 = 100%
Li et al., 2011	5	30 (total 30 min, 15 min for each foot)	1	5 (2.5 hours)	Not reported	Not reported	32/34 = 94%
Bakir et al., 2018	1	60 (total 60 min, 30 min for each foot)	6	6 (6 hours)	Not reported	Not reported	30/31 = 96%
Unal et al., 2016	2	30 (total 30 min, 15 min for each foot)	4	8 (4 hours)	Not reported	Not reported	35/35 = 100%
Zengin et al., 2018	2	30 (total 30 min, 15 min for each foot)	8	16 (8 hours)	Not reported	Not reported	84/88 = 95%
Rambod et al., 2019	5	30 (total 30 min, 15 min for each foot)	1	5 (2.5 hours)	No side effect	Not reported	36/36 = 100%
Malekshahi et al., 2018	3	10 (totally 10 min, 5 min for each foot)	4	12 (2 hours)	Not reported	Not reported	40/40 = 100%
Oshvandi et al., 2014	2	20 min (totally 20 min, 10 min for each foot)	1	2 (0.66 hours)	Not reported	Not reported	30/30 = 100%
Samarehfekri et al., 2020	3	30 min (totally 30 min, 15 min for each foot)	1	3 (1.5 hours)	No side effect	1 week after intervention	25/26 = 96%
Toygar et al., 2020	3	30 min (totally 30 min, 15 min for each foot)	1	3 (1.5 hours)	Not reported	Not reported	33/33 = 100%
Bahrami et al., 2019	1	20 min (totally 20 min, 10 min for each foot)	1	1 (0.33 hours)	No side effect	Not reported	45/45 = 100%
Noh et al., 2019	3	30 min (totally 30 min, 15 min for each foot)	6	18 (9 hours)	Not reported	Not reported	32/33 = 96%
Mahdavipour et al., 2019	2	30 min (totally 30 min, 15 min for each foot)	6	12 (6 hours)	Not reported	2 months after intervention	45/50 = 90%
Soheili et al., 2017	2	40 min (totally 40 min, 20 min for each foot)	4	8 (5.33 hours)	Not reported	Not reported	25/25 = 100%
Vardanjani et al., 2013	1	30 min	1	1 (0.5 hours)	Not reported	Not reported	50/50 = 100%
Bagheri-nesami et al., 2014	4	20 min (totally 20 min, 20 min for left foot)	1	4 (1.33 hours)	Not reported	Not reported	40/40 = 100%
Khaledifar et al., 2017	1	30 min (totally 30 min, 15 min for each foot)	1	1 (0.5 hours)	Not reported	Not reported	25/25 = 100%
Saatsaz et al., 2016	1	-	1	1 (—)	Not reported	90 min after foot massage	52/52 = 100%
Pasyar et al., 2018	1	10 (total 10 min, 5 min for each foot)	1	1 (0.16 hours)	Not reported	2 hours after foot massage	33/33 = 100%
Koras et al., 2019	1	40 (total 40 min, 20 min for each foot)	1	1 (0.66 hour)	Not reported	90 min after foot massage	85/85 = 100%
Eguchi et al., 2016	3	45 min	4	12 (9 hours)	No side effect	Not reported	27/27 = 100%
Ozturk et al., 2018	3	20 (total 20 min, 10 min for each foot)	1	3 (1 hour)	Not reported	Not reported	32/32 = 100%
Ramezanibadr et al., 2018	1	20 min	1	1 (0.33 hour)	Not reported	1 hour after foot reflexology	50/50 = 100%
Shahsavari et al., 2017	1	30 min	1	1 (0.5 hour)	Not reported	Not reported	40/40 = 100%
Abbaszadeh et al., 2018	4	30 (total 30 min, 15 min for each foot)	1	4 (2 hours)	Not reported	Not reported	40/40 = 100%
Levy et al., 2020	1	30 (total 30 min)	1	1 (0.5 hour)	No side effect	Not reported	99/99 = 100%

**Table 3:** Risk of the methodological bias score of included studies.

Authors, year	Random sequence generation (selection bias)	Allocation concealment (selection bias)	Binding of participants and personnel (performance bias)	Blinding of outcome assessment (detecting bias)	Incomplete outcome data (attrition bias)	Selective reporting bias (reporting bias)	Other bias
Valizadeh, 2015	U	U	H	U	L	L	H
Li, 2011	L	U	H	H	L	L	U
Bakir, 2018	U	U	U	U	L	L	H
Unal, 2016	U	U	U	U	L	L	H
Zengin, 2018	L	U	U	U	L	L	U
Rambod, 2019	L	L	H	L	L	L	U
Malekshahi, 2018	U	U	U	U	U	L	H
Oshvandi, 2014	L	L	H	U	L	L	U
Samarehfekri, 2020	L	L	U	U	L	L	U
Toygar, 2020	L	U	L	L	L	L	U
Bahrami, 2019	L	L	H	U	L	L	U
Noh et al., 2019	L	U	H	U	L	L	U
Mahdavipour et al., 2019	U	U	H	U	L	L	U
Soheili et al., 2017	L	U	H	U	L	L	U
Vardanjani et al., 2013	L	U	H	U	L	L	H
Bagheri-nesami et al., 2014	L	U	U	U	L	L	U
Khaledifar et al., 2017	U	U	U	U	L	L	H
Saatsaz et al., 2016	L	U	H	U	L	L	H
Pasyar et al., 2018	L	L	H	U	L	L	H
Koras et al., 2019	U	U	H	U	L	L	H
Eguchi et al., 2016	U	U	U	U	L	L	U
Ozturk et al., 2018	L	U	H	U	L	L	U
Ramezanibadr et al., 2018	L	U	H	U	L	L	U
Shahsavari et al., 2017	L	U	H	U	L	L	U
Abbaszadeh et al., 2018	L	L	U	L	L	L	U
Levy et al., 2020	L	U	H	L	L	L	U

H: high risk, L: low risk, U: unclear.

**Table 4:** Overall effect size of foot reflexology intervention for an adult.

		Effect size	95% CI		Null hypothesis		Heterogeneity		
					Two-tailed test				
	Sample size (studies)	Hedge's *g*	Lower	Upper	*Z* value	*P* Value	*Q* value	*P* value	*I* ^2^

Depression	4	−0.921	−1.246	−0.595	−5.542	<0.001	5.42	0.143	44.74
Anxiety	16	−1.237	−1.682	−0.791	−5.435	<0.001	217.41	<0.001	93.10
Sleep quality	10	−1.665	−2.361	−0.970	−4.692	<0.001	144.87	<0.001	93.78

*P* values > 0.001 were rounded to two digits. CI, confidence interval.

**Table 5:** Mean effect sizes and moderator analyses of foot reflexology intervention.

	Parameter	Results	Effect sizes (Hedges' *g*)	95%CI
Anxiety	Categorical moderators			
	Outcome measurement tool			
	STAI	9	−1.534	−2.332, −0.736
	Others	7	−0.894	−1.241, −0.547
	Reflexology before/after			
	Surgical intervention			
	Before	5	−1.409	−2.083, −0.735
	After	5	−1.745	−3.066, −0.427
	Intervention type			
	1 time intervention	9	−1.553	−2.190, −0.915
	>1 time intervention	6	−0.849	−1.471, −0.226
	Surgical intervention type			
	Cardiovascular intervention	5	−1.060	−1.652, −0.467
	Other surgery	5	−2.340	−3.485, −1.195
	Random sequence generation			
	High/unclear risk	3	−2.401	−4.737, −0.064
	Low risk	13	−0.970	−1.275, −0.666
	Allocation concealment			
	High/unclear risk	12	−1.271	−1.812, −0.730
	Low risk	3	−1.102	−1.668, −0.536

Sleep quality	Outcome measurement tool			
	PSQI	7	− 2.021	−2.931, −1.112
	Others	3	− 0.853	−1.158, −0.548
	Participant		−	
	Hemodialysis group	2	2.850	−4.104, −1.596
	Nonhemodialysis group	8	−1.375	−2.119, −0.632
	Random sequence generation			
	High/unclear risk	4	−2.032	−3.033, −1.031
	Low risk	6	−1.424	−2.395, −0.454
	Allocation concealment			
	High/unclear risk	7	−2.085	−2.913, −1.257
	Low risk	3	−0.686	−0.981, −0.390

Anxiety	Parameter	Results	Slope	95% CI
	Continuous moderators			
	Mean age	11	−0.155	−1.790, 1.480
	Total length in one time	8	−0.126	−2.217, 1.966

Sleep quality	Mean age	8	0.035	−0.049, 0.056
	Total length of time	10	−0.346	−0.568, −0.124
	Duration	10	−0.256	−0.466, −0.046

## Data Availability

The data used to support the findings of this study are included within the article.
